# PET/CT uncovers cranial giant cell arteritis

**DOI:** 10.1186/s41824-021-00114-1

**Published:** 2021-10-21

**Authors:** Tamer Anati, Michal Hoffman Ben Shabat

**Affiliations:** 1 Department of Nuclear Medicine, Rabin Medical Center, Petah Tikva, Israel; 2 Department of Internal Medicine, Rabin Medical Center, Petah Tikva, Israel

**Keywords:** [18F]FDG PET/CT, Giant cell arteritis, Temporal, cGCA

## Abstract

**Background:**

Giant cell arteritis (GCA) is an inflammation of large and medium sized vessels, mainly affecting people over 50 years of age. Diagnosis needs to be made quickly to prevent complications. Steroids treatment should be started once diagnosis is made.

**Case presentation:**

Here we reported a case of cranial GCA in a 82-year-old man. [18F]FDG PET/CT imaging demonstrated higher FDG uptake in medium sized and cranial vessels. Glucocorticoid treatment was started, followed by a rapid and marked improvement of symptoms and inflammatory markers.

**Conclusions:**

This case report supports the role of PET/CT hybrid imaging as a useful noninvasive tool in the evaluation of cranial GCA.

## Background

Giant cell arteritis, also known as temporal arteritis is categorized as a vasculitis of large and medium-sized vessels because it can involve the aorta and great vessels. Systemic symptoms are common in GCA and vascular involvement can be widespread. It is the most common idiopathic systemic vasculitis and affects mainly older adults, peak incidence between ages 70 and 79. Symptoms onset tends to be subacute and nonspecific. Treatment with glucocorticoids should be promptly once the diagnosis is made, to prevent visual loss. The diagnosis of GCA is usually based on histopathology or imaging exams. Histopathologic evidence of GCA is most often acquired by invasive temporal artery biopsy (TAB), histopathologic evidence of GCA is most often acquired by invasive temporal artery biopsy (TAB), it has been reported that false-negative results (10–61%) may occur, due to the patchy involvement of the artery—“skip lesions” (Ashton-Key and Gallagher 1992; Gonzalez-Gay et al. 2001; Oh et al. 2018). Ultrasound with Doppler is another diagnostic tool, but there is the absence of extensive experience. Other temporal artery imaging such as MRI angiography has poor sensitivity. Another diagnostic tool, as will be detailed in this case study, is PET/CT.

## Case presentation

**Fig. 1:**
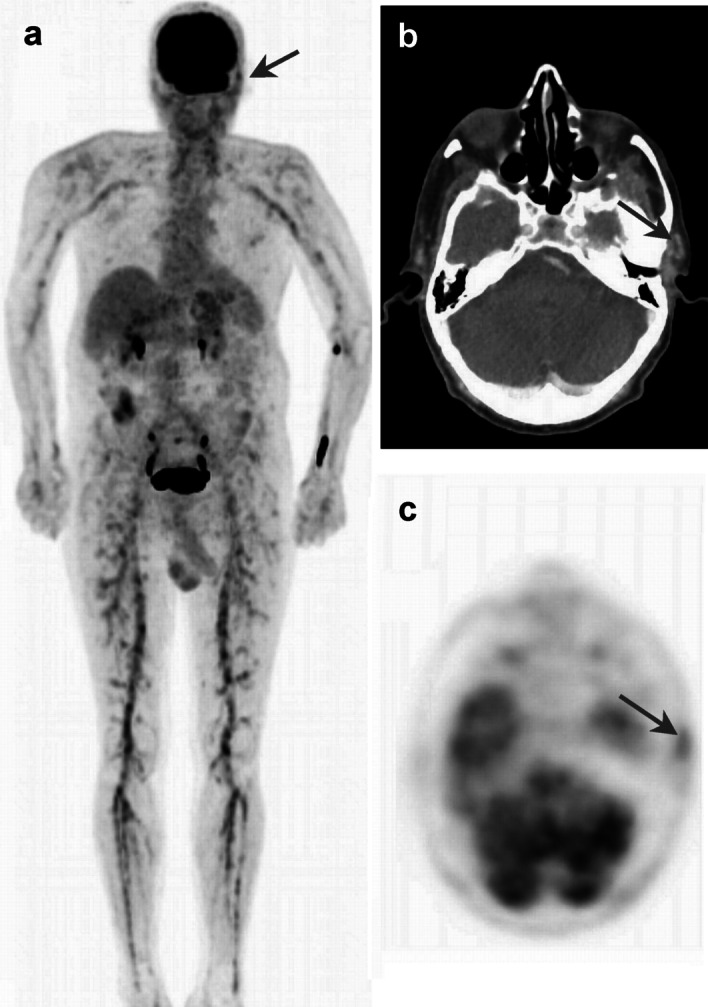
Total body [18F]FDG PET/CT was performed; coronal maximal intensity projection (MIP) image (**a**) showed increased FDG uptake of moderate-intensity along medium-sized extra-cranial (maxillary, and superficial temporal arteries), and cranial vessels; corresponding axial contrast-enhanced CT and [18F]FDG-PET (**b, c**) showed involvement of the left temporal artery with pathological tracer uptake (arrow), indicating active disease

A 82-year-old man with a medical history of Dyslipidemia, Hypertension, and glaucoma, presented with a 3-week history of fever of unknown origin and a rash. Physical examination was notable for erythematous papular rash over his trunk, fatigue and weight loss was recorded. Laboratory studies showed hemoglobin levels of 7.6 g/dL (reference range 14–17 g/dL) and thrombocytosis of 600·10^3^/µL (reference range 150–450 10^3^/µL). Ferritin levels of 1300 ng/milliliter (reference range 22–277 ng/ml) and ESR level of 120 mm/h (reference range < 20 mm/h). Serological tests for infectious agents as well as rheumatoid panels were all non-diagnostic. To support the diagnosis of FUO; PET/CT was done; the patient was prepared according to known recommendations (Slart et al. 2018). PET/CT showed involvement of cranial and extra-cranial vessels including superficial temporal, maxillary, vertebral and occipital arteries, as well as medium size vessels such as subclavian, brachial, femoral, and tibial arteries (Fig. 1). Despite the high physiologic FDG uptake in the brain, we noted that temporal artery and bilateral upper and lower extremity arteries had higher uptake than the surrounding tissue, a grade 2, and higher uptake than the liver’s, meaning grade III. SUVmax was 4.1 left temporal artery, 4.3 in axillary and brachial arteries, and 7.5 in femoral, popliteal and tibial arteries. Thoracic aorta was not significantly involved, SUVmax 3.5 (Nielsen et al. 2019; Stellingwerff et al. 2015; Slart et al. 2018; Nienhuis et al. 2020); Thus, a GCA diagnosis was presumed. Despite the abscess of jaw claudication and headaches, an ultrasound was done due to confirm the diagnosis however US and cytological findings of the temporal arteries were non-remarkable of the presumption of skips lesions. Based on the widespread distribution and typical pattern on PET/CT, a diagnosis of giant cell arteritis was made. Glucocorticoid treatment was started, followed by a rapid and marked improvement of symptoms and inflammatory markers.

## Results

The European League Against Rheumatism (EULAR) recommended diagnosing large vessel vasculitis (LVV) on clinical grounds and on diagnostic imaging (Dejaco et al. 2018). 83% sensitivity and 75% specificity was reveled by visual analysis while a 79% sensitivity and a 92% specificity were found when measuring SUVmax in the cranial arteries (Nienhuis et al. 2020). Thus [18F]FDG PET/CT hybrid imaging proved to be a useful and noninvasive tool in diagnostic evaluation of inflammatory conditions such as GCA. However additional randomized studies are needed to support this.

## Conclusions

This case report supports the role of PET/CT hybrid imaging as a useful noninvasive tool in the evaluation of cranial GCA.

## Data Availability

Data sharing is not applicable to this article as no datasets were generated or analyzed during the current study.

## Abbreviations

GCA: Giant cell arteritis
cGCA: Cranial giant cell arteritis
LVV: Large vessel vasculitis
TAB: Temporal artery biopsy
PET: Positron emission tomography
CT: Computed tomography
MIP: Maximum intensity projection
[18F]FDG: 18 Fluroide-Fulorodeoxyglucose
SUVmax: Maximum standardized uptake value
